# DiCyc: GAN-based deformation invariant cross-domain information fusion for medical image synthesis

**DOI:** 10.1016/j.inffus.2020.10.015

**Published:** 2021-03

**Authors:** Chengjia Wang, Guang Yang, Giorgos Papanastasiou, Sotirios A. Tsaftaris, David E. Newby, Calum Gray, Gillian Macnaught, Tom J. MacGillivray

**Affiliations:** a BHF Centre for Cardiovascular Science, University of Edinburgh, Edinburgh, UK; b National Heart and Lung Institute, Imperial College London, London, UK; c Edinburgh Imaging Facility QMRI, University of Edinburgh, Edinburgh, UK; d Institute for Digital Communications, School of Engineering, University of Edinburgh, Edinburgh, UK; e Centre for Clinical Brain Sciences, University of Edinburgh, Edinburgh, UK

**Keywords:** Information fusion, GAN, Image synthesis

## Abstract

Cycle-consistent generative adversarial network (CycleGAN) has been widely used for cross-domain medical image synthesis tasks particularly due to its ability to deal with unpaired data. However, most CycleGAN-based synthesis methods cannot achieve good alignment between the synthesized images and data from the source domain, even with additional image alignment losses. This is because the CycleGAN generator network can encode the relative deformations and noises associated to different domains. This can be detrimental for the downstream applications that rely on the synthesized images, such as generating pseudo-CT for PET-MR attenuation correction. In this paper, we present a deformation invariant cycle-consistency model that can filter out these domain-specific deformation. The deformation is globally parameterized by thin-plate-spline (TPS), and locally learned by modified deformable convolutional layers. Robustness to domain-specific deformations has been evaluated through experiments on multi-sequence brain MR data and multi-modality abdominal CT and MR data. Experiment results demonstrated that our method can achieve better alignment between the source and target data while maintaining superior image quality of signal compared to several state-of-the-art CycleGAN-based methods.

## Highlights

• Novel DiCyc architecture filters out domain-specific deformations.
• The deformable layers are modified for less parameters and faster convergence.
• Expectation–maximization training with 2-forward-1-backward back propagation.
• New cycle-consistency and image alignment losses with less conflicted gradients.
• An ablation study visualizes the trade-off between image alignment and quality.

## 1 Introduction

Multi-modal medical imaging, i.e. acquiring images of the same organ or structure using different imaging techniques (or modalities) that are based on different physical phenomena, is increasingly used towards improving clinical decision-making. However, collecting data from the same patient using different imaging techniques is often impractical, due to, limited access to different imaging devices, additional time needed for multiple scanning sessions, and the associated cost. This makes cross-domain medical image synthesis a technology that is gaining popularity. We use the term “domain” herein to refer to different imaging modalities, contrast and parametric configurations, for example, for magnetic resonance imaging (MRI). We present a method, called DiCyc, that can perform cross-domain medical image synthesis by learning from non-paired data, thus taking advantage of multiple sources of images, but due to new network architectures it is immune to the presence of deformations inherent to some medical imaging techniques.

Cross-domain image synthesis^2^ has been used to impute incomplete information in standard statistical analysis [1], [2], to predict and simulate developments of missing information [3], or to improve intermediate steps of analysis such as registration [4], information fusion [5], [6], [7], segmentation [8], [9], [10], atlas construction [11], [12] and disease classification [13], [14]. These methods map between MRI, computed tomography (CT), positron emission tomography (PET) and ultrasound imaging from one domain to another. Our main motivation is to synthesize CT images or a particular MR image contrast from multi-sequence MR data. We require the synthesized data should be usable for further medical applications, for example, using synthesized or “pseudo” CT images to improve PET-MR attenuation correction [15], [16], [17], [18], [19]. Using MRI to achieve attenuation correction of PET data can be a disadvantage as, unlike CT, the MR signal is not physically related to attenuation of x-rays in tissue. To overcome this, pseudo-CT generated from corresponding MR could be used to compute a map of linear attenuation coefficients (μ-map) and used for attenuation correction of the PET data acquired on a PET-MRI scanner [20]. This requires mapping of the geometric correspondences between CT and MR.

**Fig. 1:**
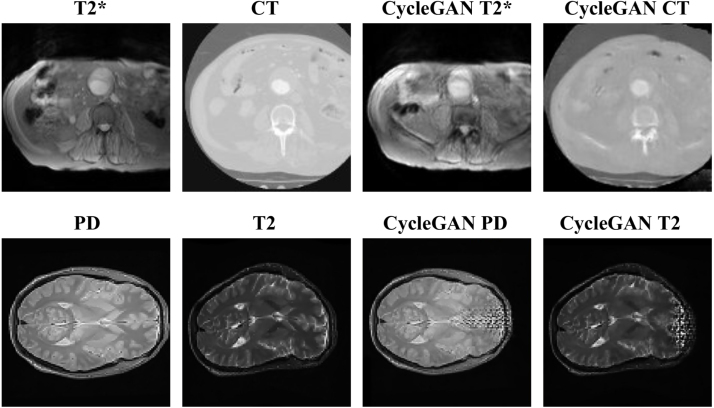
Example of cross-domain synthesis using vanilla CycleGAN. The first row shows the results obtained from cross-modality abdominal MR-CT data; the second row shows the results of multi-sequence brain data with a synthesized deformation. Both cases demonstrate a reproduction of “domain-specific deformation” in the synthesized output.

Learning a contextual correspondence between domains requires not only paired, but well-aligned training data. Such data can be generated by a reliable automatic or manual registration algorithm. As a result, the vast majority of cross-modality image synthesis methods are solely applicable to, or evaluated on brain image data [1], [2], [4], [8], [13], [16], [17], [18], [19], [21], [22], [23], [24], [25], [26], [27], [28], [29], due to the low geometric variance across different imaging modalities for this particular organ. For other organs, most methods require that the data be aligned by affine transformations or small deformations [3], [9], [25], [30], [31], [32], [33]. However, very distinct geometric variances may occur among these data. Nonlinear geometric variances are often associated with different modalities, such as those caused by the shape of imaging bed, the field of view and the axial location planning (captured in Fig. 1). We refer to these as “domain-specific deformations”, the presence of which can compromise the quality of the synthesis. This depends on whether the network can learn the mapping sufficiently by being invariant to the presence of deformations (which depends on landing on an ideal local minimum of the loss), or whether pre-processing has removed the deformation due to successful registration (which is not always feasible and cannot deal with large field of view differences).

Methods that allow training with unregistered or unpaired data have recently been proposed [34]. Most state-of-the-art methods use deep convolutional neural networks (CNN) as the image generator within a generative adversarial network (GAN) framework [35]. GAN can represent sharp and even intractable probability densities through a nonparametric approach. It has been widely used in medical image analysis, especially for data augmentation and multi-modality image translations, due to its ability of dealing with domain shift [36]. A popular direction for cross-domain image synthesis is to leverage CycleGAN [37] into the training process. Previous studies have shown that CycleGAN can be trained with unpaired brain data [22], [28]. However, CycleGAN can mistakenly encode domain-specific deformations as domain specific features and reproduce the deformations in the synthesized output. Fig. 1 demonstrates two examples. The first row shows a synthesis performed between abdominal CT and T2*-weighted MR, while the second row gives an example of T2-weighted and proton density brain MR with a simulated deformation. In both cases, the deformations specific to the input sources are reproduced by CycleGAN in the output. For applications, such as, attenuation correction where voxel-wise attenuation coefficients are computed, domain-specific deformations should be discarded whilst contextual information relating to the cross-domain appearance of anatomical features and organs is retained.

**Fig. 2:**
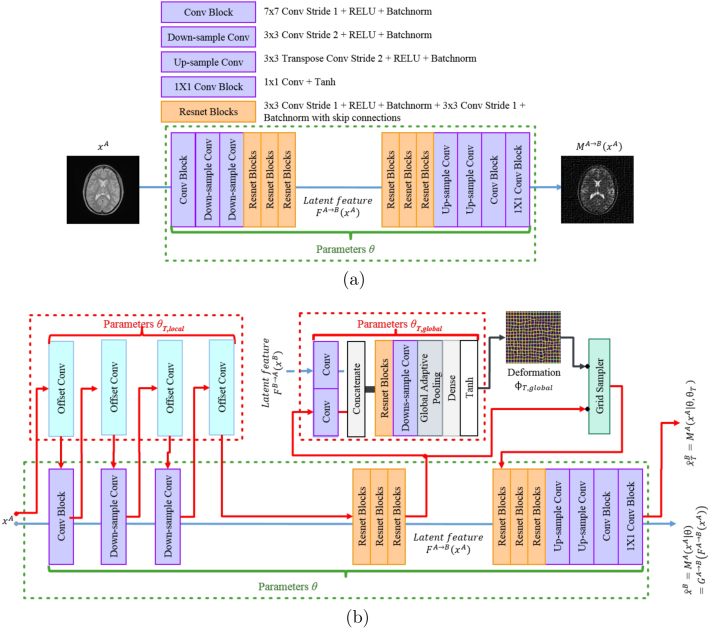
Comparison of network architectures between CycleGAN and DiCyc. (a) The generator of CycleGAN model used in the original CycleGAN, which is a normal CNN. (b) shows the DiCyc generator network. A deformation convolution layer is inserted in each block before the stack of the Resnet blocks to model the local deformation, parameterized by $\theta_{T,local}$. The global non-linear distortion is modeled using thin-plate-spline (TPS) generated by a spatial transformation subnetwork, parameterized by $\theta_{T,global}$. Details of the modified deformable convolution is shown in Fig. 4(c). The blue arrows represent the CycleGAN forward pass. The additional forward pass introduced by the deformable convolutional layers is represent as red arrows. (For interpretation of the references to color in this figure legend, the reader is referred to the web version of this article.)

Recently, several modifications of the vanilla CycleGAN have been proposed, to enhance the alignment between data from the source and target domain using an additional image alignment measure [30], [32], [38]. However, the additional image alignment loss conflicts with the original loss function in CycleGAN. The synthesized data in which the domain-specific deformations are reproduced will lead to a lower adversarial loss (of the discriminator in GAN). At the same time, the reproduced deformations harm the alignment between the source and the synthesized data, which leads to higher alignment loss. As a result, the synthesized data cannot be aligned to the source data particularly well while maintaining a good quality of signal. To address this issue, we propose the deformation invariant CycleGAN model, or DiCyc. Fig. 2 presents the structural differences between the vanilla CycleGAN and the proposed DiCyc generator networks. We introduce a global transformation model and modified layers of the deformable convolutional network (DCN) into the CycleGAN image generator and propose to the use of a novel image alignment loss based on normalized mutual information (NMI). We evaluate the proposed method using both a publicly available multi-sequence brain MR dataset and our private multi-modality (CT, MR) abdominal dataset. DiCyc displayed better ability to handle disparate imaging domains and to generate synthesized images aligned with the source data whilst keeping comparable quality of the output, compared to state-of-the-art models. Furthermore, the ablation experiment demonstrated that, unlike in the state-of-the-art models, the image alignment loss and the GAN loss were minimized together during training without conflicts in DiCyc.

The main contributions of this paper are as follows:

1. We propose a novel DiCyc architecture using a global transformation network and modified deformable convolution layers in between normal convolution layers to address the problem of domain-specific deformations. The deformable layers are modified to have less parameters and offer faster convergence.
2. Rather than the classical “1 forward pass, 1 backward pass” training routine, we designed a new expectation–maximization training procedure where each training iteration includes two distinct forward passes (shown as the blue and red arrows in Fig. 2b) and one single backward pass.
3. We designed a novel cycle-consistency loss and an image alignment loss for information fusion. These losses, together with the new training procedure, address the conflict observed between image alignment loss and the discriminative loss of GAN.
4. We visualized and quantitatively assessed the influence of the domain-specific deformation. We demonstrated the negative effects of the conflict between the image alignment loss and GAN loss in experiments using simulated brain data and realistic abdominal data, and visualized these effects on model convergence in our ablation study.

The paper is organized as follows. Section 2 reviews previous and related techniques. Section 3 gives details of the DiCyc network architecture and the associated loss function. Experiments and datasets used are described in Section 4. The results and discussion are presented in Section 5. Conclusions are given in Section 6.

## 2 Related works

### 2.1 Non-CycleGAN models

Typically, most image synthesis methods build up a mapping function from a source to a target domain using paired and pre-registered data. The mapping can be constructed by learning a regression or a dictionary from a collection of patches or feature examples as in [16], [17], [19], [39], [40]. Another conventional approach is to build an atlas for each domain using registration, such as modality propagation [41], [42], [43]. The prediction is given by mapping between atlases. Along with the rise of deep learning in recent years, neural networks have been used as the cross-domain regressor. For example, the Location Sensitive Deep Network (LSDN) [27] uses a CNN to map the location-dependent patch information between domains. In [26], [29], a GAN framework are used to learn the mapping function with context-aware measure based on gradient difference loss. Similarly, [44] uses conditional GAN to synthesize lung histology images. An early method using unpaired data is proposed in [34] where training with unpaired data was addressed as an *unsupervised* approach. It uses mutual information (MI) to select the best corresponding image patches from unpaired cross-domain data, and maximizes a mixture of global MI and local spatial consistency to synthesize multi-sequence brain MR data. This work uses a preprocessing procedure [41] which includes a registration step. Another approach similar to [34] is to construct a dictionary from patches or image pairs [19], [39]. In [45], an algorithm using Weakly-coupled And Geometry (WAG) co-regularized joint dictionary learning is proposed, which learns the patch correspondence from partially unpaired data. Yet, this method was only evaluated using brain images with small geometric variances. A natural strategy in current deep learning based medical image synthesis methods is to model the latent features to arbitrary distributions. For example, [46] assumes the latent features follow a mixed Gaussian distribution, but this method was only evaluated on multi-contrast CT images for segmentation tasks. This paper concentrates on more general synthesis problems between multi-sequence MR data or multi-modal MR and CT data.

### 2.2 CycleGAN-based methods

CycleGAN was first applied to cross-domain medical image synthesis in [31] and [28] for co-synthesis of CT and MR cardiac and brain data respectively. Both works hint at the influence of deformation affecting results and so removed such artifacts by regularizing the problem through adding additional information (e.g. segmentation masks) [31], and by co-registration [28]. Similarly, in [11], [25], [30], [31], [47], the performance of image synthesis networks can be enhanced when jointly trained for segmentation tasks. However, these models require extra manual annotations or registration. Without this requirement, many methods integrate image similarity measures into the GAN loss, for matching the same structure across different domains. For example, [48] introduced a structure-consistency loss based on the modality independent neighborhood descriptor (MIND) [49]. It has been demonstrated that this structure-constrained CycleGAN can deal to some extent with unregistered multi-modal MR and CT brain data. A similar gradient-consistency loss, based on the normalized gradient cross correlation (GCC), is used in [32] for the same purpose. This method has been evaluated using unpaired but pre-registered, multi-modal MR and CT hip images. However, as discussed in Section 1, there is a conflict between the image similarity based losses and the CycleGAN discriminative loss. One potential solution of this problem is to factorize the latent representations into domain-independent semantic features and domain-dependent appearance features, and explicitly filter out the relative spacial deformation between the source and target data [50], [51], [52]. This work extends this idea for larger deformations and wider range of domains.

## 3 Method

### 3.1 Notation and background

Our goal is to generate synthesized CT or MR data to help post-processing of the source data. For example, a pseudo-CT μmap applicable to PET-MR attenuation correction without registering the synthesized data to the source.

We assume that we have $n^{A}$ images $x^{A}\in X^{A}$ from domain $X^{A}$, and $n^{B}$ images $x^{B}\in X^{B}$ from domain $X^{B}$. For a source image $x^{A}$, a generator, $M^{A\to B}$, is trained to generate a synthesized image ${\overset{ˆ}{x}}^{B}=M^{A\to B}\left(x^{A}\right)$. Following the GAN setup, $M^{A\to B}$ and a discriminator $D^{B}$ ares trained to solve the min–max problem of the GAN loss $L_{GAN}\left(M^{A\to B},D^{B},X^{A},X^{B}\right)$. For brevity, we let $L_{GAN}^{A\to B}$ denote the GAN loss. $M^{A\to B}$ maps the data from $X^{A}$ to $X^{B}$ while $D^{B}$ is trained to distinguish whether an image is real or synthesized. Accordingly, for synthesis from $X^{B}$ to $X^{A}$, there are a $M^{B\to A}$, a $D^{A}$, and a GAN loss $L_{GAN}^{B\to A}$. The vanilla CycleGAN framework consists of two symmetric sets of generators $M^{A\to B}$ and $M^{B\to A}$ act as mapping functions applied to a source domain, and two discriminators $D^{B}$ and $D^{A}$ to distinguish real and synthesized data for a target domain [37]. The *cycle consistency* loss $L_{cyc}\left(M^{A\to B},D^{A},M^{B\to A},D^{B},X^{A},X^{B}\right)$, or $L_{cyc}^{A,B}$, is used to keep the cycle-consistency between the two sets of networks [37]. This gives CycleGAN the ability to deal with unpaired data. Then the loss of the whole CycleGAN framework $L_{CycleGAN}$ is $L_{CycleGAN}=L_{GAN}^{A\to B}+L_{GAN}^{B\to A}+\lambda_{cyc}L_{cyc}^{A,B}$. Recent improvements of CycleGAN [32], [48] add an image alignment term $L_{align}^{A,B}$ to $L_{CycleGAN}$ which becomes

(1) $$L_{CycleGAN,align}=L_{CycleGAN}+\lambda_{align}L_{align}^{A,B}=L_{GAN}^{A\to B}+L_{GAN}^{B\to A}+\lambda_{cyc}L_{cyc}^{A,B}+\lambda_{align}L_{align}^{A,B},$$

where $\lambda_{align}$ is the weight used to balance the effects of $L_{align}^{A,B}$ and $L_{CycleGAN}$. As discussed in Section 1, this causes the conflict between quality of synthesis images and source-target image alignment. The later parts of this section present the detailed analysis of this problem and our DiCyc solution.

### 3.2 Dicyc architecture

Adding the alignment loss $L_{align}$ makes cross-domain image synthesis a multi-task learning problem: M is trained for image synthesis while aligning the source and synthesized images. Because the relative deformation, ϕ, between the source and target training images are partially domain specific, this information is encoded by the discriminator D. Note that $L_{align}$ and $L_{CycleGAN}$ in existing methods [32], [48] are both works on the source image x and the synthesized image M(x). Assuming M(x) is well aligned to x, and ${\overset{ˆ}{x}}_{T}^{B}=M\left(x\right)\circ \phi$ is identical to the target image, even when both images have the same image quality, it is always true that

(2) $$L_{GAN}\left(D^{*}\left(x\right),D^{*}\left(M\left(x\right)\right)\circ \phi \right)<L_{GAN}\left(D^{*}\left(x\right),D^{*}\left(M\left(x\right)\right)\right)$$

for an optimal discriminator $D^{*}$. At the same time,

(3) $$L_{align}\left(x,M\left(x\right)\right)>L_{align}\left(x,M\left(x\right)\circ \phi \right).$$

As a result, $L_{GAN}$ and $L_{align}$ lead to gradients with opposite directions: $sgn\left(\nabla_{\theta }L_{GAN}\right)\neq sgn\left(\nabla_{\theta }L_{align}\right)$ where θ is the network parameters. Any choice of the hyperparameter $\lambda_{align}>0$ or data augmentation for D will cause a trade-off between the image quality and data alignment.

To solve the problem of inverse gradients, we model the deformation ϕ using a separated set of parameters $\theta_{T}$. For example, in the A→B process, $M^{A\to B}$ outputs two synthesized images: one undeformed image aligned to the source:

(4) $${\overset{ˆ}{x}}^{B}=M^{A\to B}\left(x^{A}\right)=M^{A\to B}\left(x^{A}|\theta^{A\to B}\right),$$

and one deformed image that is identical to the target:

(5) $${\overset{ˆ}{x}}_{T}^{B}=M_{T}^{A\to B}\left(x^{A}\right)=M^{A\to B}\left(x^{A}|\theta^{A\to B},\theta_{T}^{A\to B}\right).$$

As shown in Fig. 1, the relative deformation between the source and target domains can be seen as a combination of a global and a local transformation, thus $\phi =\phi_{global}\circ \phi_{local}$. The corresponding transformation parameters $\theta_{T}=\left\{\theta_{T,global},\theta_{T,local}\right\}$ are modeled by in different subnetworks in the DiCyc generator (Fig. 2b).

We split the generator M into three subnetworks: an encoder, F, a decoder G and a transformer T. T estimates the global transformation $\phi_{global}$, parameterized by $\theta_{T,global}$. In previous CycleGAN based methods parameterize F and F with image synthesize parameters θ. In our DiCyc model, the generator F also estimates the local deformations, parameterized by $\theta_{T,local}$ which is introduced by a series of deformable convolutional layers. As a results, F also produced two versions of latent features: the undeformed feature map F(x)=F(x|θ) and the locally deformed feature $F_{T}\left(x\right)=F\left(x\right)\circ \phi_{local}=F\left(x|\theta ,\theta_{T,local}\right)$.

### 3.3 Global deformation

The global transformer T has a similar structure with the thin-plate-spline (TPS) based STN. As shown in Fig. 2b, in the A→B process, the global deformation is calculated by:

(6) $$\phi_{global}^{A\to B}=T^{A\to B}\left(Conv\left(z^{A\to B}\right)⊕Conv\left(z^{B\to A}\right)\right),$$

where $z^{A\to B}$ and B→A are latent features given by the encoders $F^{A\to B}$ and $F^{B\to A}$, and ⊕ represents the concatenation operation. Specifically, a regular grid of 6 × 6 control points $t^{B}=\left\{t_{i}^{B}|i\in \left\{1,\ldots ,36\right\}\right\}$ is placed on the latent feature maps of $x^{B}$. $T^{A\to B}$ outputs the coordinates of corresponding points $t^{A}$ on features of $x^{A}$. TPS maps the deformation decided by $t^{A}$ and $t^{B}$ using an interpolation function Φ. Φ has a form of:

(7) $$t^{B}=\Phi \left(t^{A}\right)=c+At+W^{T}s\left(t\right),$$

where t is regular image grid and W is the weights assigned to the control points. c and A define the affine transformation between $t^{A}$ and $t^{B}$. s is defined as:

(8) $$s\left(r\right)={\left(\delta \left(t-t_{1}\right),\delta \left(t-t_{2}\right),\ldots ,\delta \left(t-t_{36}\right)\right)}^{T},$$

where σ is a radial basis kernel has the form of:

(9) $$\delta \left(r\right)=r^{2}\log r.$$

Note that the transformer T uses a normalized grid where the coordinates t∈[−1,1].

**Fig. 3:**
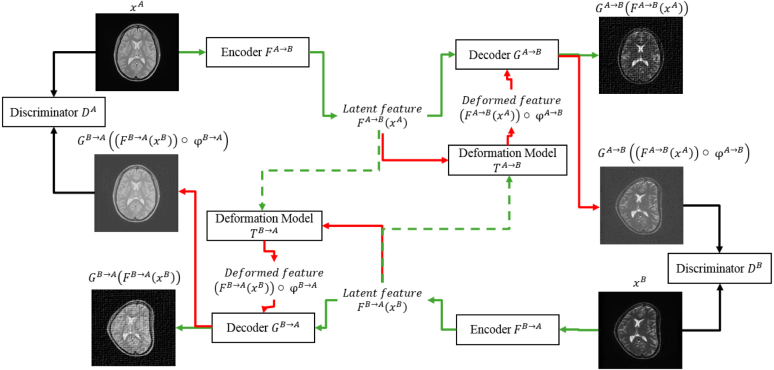
The DiCyc framework when ignoring local deformation being trained for cross synthesis of PD-weighted (A) and T2-weighted (B) images. The A→B process is shown by the green arrow and the B→A process is shown in red. (For interpretation of the references to color in this figure legend, the reader is referred to the web version of this article.)

It has been proved that this form of interpolation function minimizes the bending energy of a surface [53], so it introduces minimal affection on image quality. Based on this analysis, for better quality of synthesis, we wish to keep the local deformation to minimum level within tiny spatial area. When ignoring the local deformation $\phi_{T,local}$, the whole DiCyc model is shown as in Fig. 3.

### 3.4 Local deformation

We use a modified DCN structure in the encoder G to model the deformation in a local neighborhood after the latent feature $z^{A\to B}$ and $z^{B\to A}$ are globally aligned. A deformable convolutional layer interpolates the input feature maps through an “offset convolution” operation, followed by a normal convolutional layer [54]. This architecture separates the information about local spatial deformation and image context into two forward passes, thus further removes the conflict introduced by $L_{align}$.

As shown in Fig. 2b, we add an offset convolutional layer (displayed in cyan) before the input convolution layer, the two down-sample convolution layers and the stack of Resnet blocks. This leads to a “lasagne-like” structure consisting of interleaved “offset convolution” and conventional convolution operations so that the spatial deformation is gradually encoded through each layer. The red and blue arrows in Fig. 2b display the computation flows for generating $F_{T}\left(x^{A}\right)$ and F(x) in the forward passes.

**Fig. 4:**
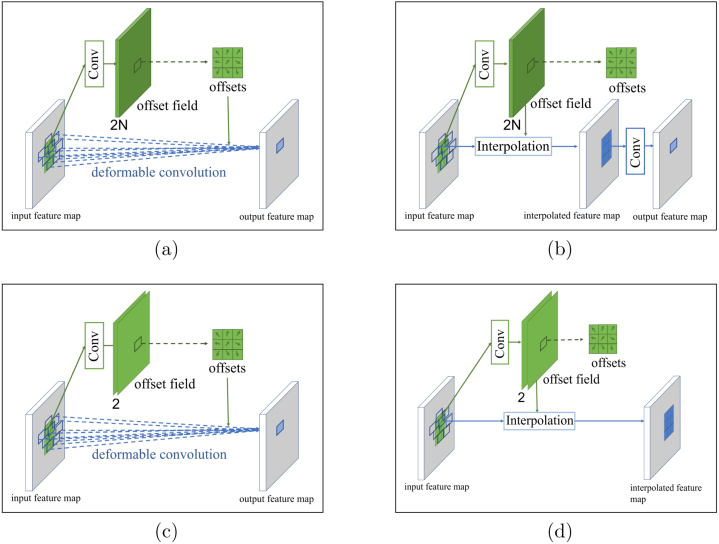
Details of the original deformable convolution and our modified version. (best viewed in color).

Fig. 4 demonstrates details of the deformable convolution and our modified version used in this work. The deformable convolution can be viewed as an atrous convolution kernel with trainable dilation rates as shown in Fig. 4a. This dilation rate varies across different locations of the input feature maps. As shown in Fig. 4b, the offset of each point in the “N-channel” input feature maps is learned by a standard convolutional operation, outputting 2N “offset maps” (a 2-D deformation for each input feature map is represented by 1 “x” and 1 “y” offset map) [54]. The N input feature maps are then interpolated using the 2N offset feature maps. These operations together are termed as “offset convolution”. A standard convolution layer is then applied to the interpolated feature map. When put together these operations form a deformable convolution operation. Designed originally for object recognition tasks, the deformable convolution operation deforms each input feature map independently. Instead, to adjust this operation to cross-domain image synthesis, our modified deformable convolution generates a uniform 2-D deformation that is valid for all input feature maps (Fig. 4c). This is equivalent to directly applying a deformation to the input image and passing it forward through the vanilla CycleGAN generator. This reduces the number of parameters in DCN to a minimum level. Fig. 4d shows our implementation of the “offset convolution”.

Combined with the global transformation, ${\overset{ˆ}{x}}_{T}^{B}=F^{A\to B}\left(F_{T}^{A\to B}\left(x^{A}\right)\circ \phi_{global}^{A\to B}\right)$ is then taken by the corresponding discriminator $D^{B}$ to compute GAN losses, and ${\overset{ˆ}{x}}^{B}=G^{A\to B}\left(F^{A\to B}\left(x^{A}\right)\right)$ is expected to be aligned with $x^{A}$.

Training DiCyc loss involves the traditional GAN loss, the cycle-consistency loss used in the original implementation of CycleGAN [37], as well as an image alignment loss and an additional cycle consistency loss introduced by the auxiliary outputs obtained from our two separated forward passes. We detail these below.

#### 3.4.1 GAN loss

For the GAN loss $L_{GAN}^{A\to B}$, the minmax game of $M^{A\to B}$ and $D^{B}$ is represented as:

(10) $$M^{A\to B*},D^{B*}=\arg\underset{D^{B}}{\min}\underset{M^{A\to B}}{\max}L_{GAN}^{A\to B},$$

where $M^{A\to B*}$ and $D^{B*}$ represents optimal generator and discriminator. Theoretically, in our DiCyc model, the loss function of $D^{B}$ is:

(11) $$L_{GAN}^{D^{B}}=E_{x∼p_{X^{B}}}\log\left(D^{B}\left(x\right)\right)+E_{x∼p_{X^{A}}}\log\left(1-D^{B}\left(M_{T}^{A\to B}\left(x\right)\right)\right),$$

where $p_{X^{A}}$ and $p_{X^{B}}$ represent the data distribution in domain $X^{A}$ and $X^{B}$. The GAN loss of generator $M^{A\to B}$ is:

(12) $$L_{GAN}^{A\to B}=E_{x∼p_{X^{A}}}\log\left(D^{B}\left(M_{T}^{A\to B}\left(x\right)\right)\right).$$

Similarly, the GAN loss of $M^{B\to A}$ is:

(13) $$L_{GAN}^{B\to A}=E_{x∼p_{X^{B}}}\log\left(D^{A}\left(M_{T}^{B\to A}\left(x\right)\right)\right).$$

#### 3.4.2 Image alignment loss

Eq. (11) can be rewritten as:

(14) $$L_{GAN}^{D^{B}}=E_{x∼p_{X^{B}}}\log\left(D^{B}\left(x\right)\right)+E_{x∼p_{{\overset{ˆ}{X}}_{T}^{B}}}\log\left(1-D^{B}\left(M_{T}^{A\to B}\left(x\right)\right)\right),$$

where $p_{{\overset{ˆ}{X}}_{T}^{B}}$ is the distribution of synthesized domain B images. $D^{B}$ is then trained to discriminate the distributions $x∼p_{X^{B}}$ and $x∼p_{{\overset{ˆ}{X}}_{T}^{B}}$ [35]. In the minmax game of the original GAN model, it has been proved that an optimal discriminator $D^{B*}=p_{X^{B}}∕\left(p_{X^{B}}+p_{{\overset{ˆ}{X}}_{T}^{B}}\right)$. Substituting this into $L_{GAN}^{D^{B}}$, it can be rewritten as:

(15) $$L_{GAN}^{D^{B}}=E_{x∼p_{X^{B}}}\log\frac{p_{X^{B}}}{p_{X^{B}}+p_{{\overset{ˆ}{X}}_{T}^{B}}}+E_{x∼p_{{\overset{ˆ}{X}}^{B}}}\log\frac{p_{X^{B}}}{p_{X^{B}}+p_{{\overset{ˆ}{X}}_{T}^{B}}}-\log4+\;E_{x∼p_{{\overset{ˆ}{X}}^{B}}}\log4=KL\left(p_{X^{B}}∥\frac{p_{X^{B}}+p_{{\overset{ˆ}{X}}_{T}^{B}}}{2}\right)+KL\left(p_{{\overset{ˆ}{X}}^{B}}∥\frac{p_{X^{B}}+p_{{\overset{ˆ}{X}}_{T}^{B}}}{2}\right)-log4=2\cdot JSD\left(p_{X^{B}}|p_{{\overset{ˆ}{X}}_{T}^{B}}\right)-log4,$$

where KL is the Kullback–Leibler divergence and JSD is the Jensen–Shannon divergence.

Let $\psi^{A}$ and $\psi^{B}$ be the spatial poses of the two images, and $\psi^{A}∼p_{\psi }^{A}$ and $\psi^{B}∼p_{\psi }^{B}$. For a pair of training images, the relation between $\psi^{A}$ and $\psi^{B}$ is:

(16) $$\psi^{B}=\psi^{A}\circ \phi^{A\to B}=\psi^{A}\circ \phi^{-B\to A}=\psi^{B}\circ \iota ,$$

(17) $$\psi^{A}=\psi^{B}\circ \phi^{B\to A}=\psi^{B}\circ \phi^{-A\to B}=\psi^{A}\circ \iota ,$$

where $\phi^{-\cdot }$ represents the inverse transformation and ι represents the identical transformation. With training data which is suffering from the domain-specific deformation, optimally trained $D^{B*}$ and $T^{A\to B*}$ will inevitably predict that $p\left(x|\psi^{A},\phi^{A\to B}\right)=p_{data}\left(x^{B}\right)$ and $p\left(x|\psi^{A},\iota \right)\in p_{fake}\left(x^{B}\right)$ even when ${\overset{ˆ}{x}}^{B}$ has comparable quality with $x^{B}$. As the GAN losses are calculated using ${\overset{ˆ}{x}}_{T}^{A}$ and ${\overset{ˆ}{x}}_{T}^{B}$, a new discriminative loss is required to predict which $p_{\psi ,\phi }$ the data is sampled from. Based on the infoGAN theory [55], we can maximize the mutual information (MI) between ϕ and x, as it can be easily proved that

(18) $$MI\left(x^{B},\psi^{A}|\phi^{A\to B}\right)=MI\left(x^{B},\psi^{B}\right)=H\left(x^{B}\right)-H\left(x^{B}|\psi^{B}\right)=JSD\left(p_{X^{B}}∥p_{\psi^{B}}\right).$$

MI yields values from 0 to +∞, which makes it difficult to be scaled and combined with other losses. Here we propose to use an image alignment loss based on NMI:

(19) $$L_{align}^{A,B}=2-NMI\left(x^{A},G^{A\to B}\left(x^{A}\right)\right)-\;NMI\left(x^{B},M^{B\to A}\left(x^{B}\right)\right).$$

Because the deformations are modeled by a separated set of parameters, this image alignment loss can be adopted with any similarity measure suitable for image registration, such as normalized mutual information (NMI) [56], normalized GCC used in [32], or MIND in [48] and [49].

#### 3.4.3 Cycle-consistency losses

The cycle-consistency loss plays a critical role for the improved performance of CycleGAN compared to a single GAN network, as it forces $M^{A\to B}$ and $M^{B\to A}$ learning mutually recoverable information from distinct domains. As in DiCyc, each generator produces an undeformed and deformed version of synthesized data, both should be cycle-consistent to encode optimal representation. This results in two cycle-consistency losses in our DiCyc model. The undeformed cycle consistency loss is defined as:

(20) $$L_{cyc}^{A,B}=∥M^{B\to A}\left(M^{A\to B}\left(x^{A}\right)\right)-x^{A}{∥}_{1}+\;∥M^{A\to B}\left(M^{B\to A}\left(x^{B}\right)\right)-x^{B}{∥}_{1},$$

and the deformation-invariant cycle consistency loss is:

(21) $$L_{dicyc}^{A,B}={\left\|M_{T}^{B\to A}\left(M_{T}^{A\to B}\left(x^{A}\right)\right)-x^{A}\right\|}_{1}+\;{\left\|M_{T}^{A\to B}\left(M_{T}^{B\to A}\left(x^{B}\right)\right)-x^{B}\right\|}_{1}.$$

### 3.5 Training procedure

Based on the discussion above, the overall loss of our DiCyc model is^3^

(22) $$L_{DiCyc}=L_{GAN}^{A\to B}+L_{GAN}^{B\to A}+\lambda_{align}L_{align}^{A,B}+\;\lambda_{cyc}L_{cyc}^{A,B}+\lambda_{dicyc}L_{dicyc}^{A,B}.$$

Treating the cycle-consistent losses as a kind of regularization, training the DiCyc model can be seen as a maximum likelihood estimation (MLE):

(23) $$\overset{ˆ}{\theta }=\arg\max\underset{\left(x^{A},x^{B}\right)}{\sum }\log p\left(\left(x^{A},x^{B}\right)|\theta \right)=\arg\max\underset{\left(x^{A},x^{B}\right)}{\sum }\log\underset{\left(\psi^{A},\psi^{B}\right)}{\sum }p\left(\left(x^{A},x^{B}\right),\left(\psi^{A},\psi^{B}\right)|\theta \right)=\arg\max\underset{\left(x^{A},x^{B}\right)}{\sum }\log\underset{\left(\psi^{A},\psi^{B}\right)}{\sum }q\left(\left(\psi^{A},\psi^{B}\right)\right)\frac{p\left(\left(x^{A},x^{B}\right),\left(\psi^{A},\psi^{B}\right)|\theta \right)}{q\left(\left(\psi^{A},\psi^{B}\right)\right)}=\arg\max\underset{x}{\sum }\log\underset{\psi }{\sum }q\left(\psi \right)\frac{p\left(x,\psi |\theta \right)}{q\left(\psi \right)}$$

where q(ψ) is an unknown distribution of the image poses. Based on Jensen’s inequality, as log(⋅) is an convex function,

(24) $$\underset{x}{\sum }\log\underset{\psi }{\sum }q\left(\psi \right)\frac{p\left(x,\psi |\theta \right)}{q\left(\psi \right)}\geq \underset{x}{\sum }\underset{\psi }{\sum }\log q\left(\psi \right)\frac{p\left(x,\psi |\theta \right)}{q\left(\psi \right)},$$

which gives a lower bound of the maximum likelihood. To make the equality established, $\frac{p\left(x,\psi |\theta \right)}{q\left(\psi \right)}=c$, where c is a constant. Thus the distribution q(ψ) is:

(25) $$q\left(\psi \right)=\frac{p\left(x,\psi |\theta \right)}{\underset{\psi }{\sum }p\left(x,\psi |\theta \right)}=p\left(\psi |x,\theta \right).$$

This MLE learning can be performed through an expectation–maximization (EM) training procedure. The “E” step estimates the distribution q(ψ) by:

(26) $$q\left(\psi_{i}\right)=q\left(\psi_{i}|\psi_{i-1},\theta_{T}^{A\to B},\theta_{T}^{B\to A}\right),$$

where $\psi_{i-1}$ is decided by the sample training data. For learning optimal global transformations, we fixed the parameters of G and F while only update the STN T. In other world, only the parameters $\theta_{global}$ are updated. In the “M” step, the two synthesized images $\overset{ˆ}{x}$ and ${\overset{ˆ}{x}}_{T}$ are calculated through two forward passes. The parameters θ are updated based on $L_{DiCyc}$.

## 4 Experiments

### 4.1 Datasets and preprocessing

**IXI dataset:** We selected two datasets for multi-sequence MR and cross-modality MR-CT data synthesis tasks. The first was the Information eXtraction from Images (IXI) dataset^4^ which provides co-registered multi-sequence skull-stripped 1.5T and 3T MR images collected from multiple sites. We used 66 proton density (PD-) and T2-weighted volumes, each volume containing 116 to 130 2D slices. For training and testing, 38 pairs and 28 pairs were used, respectively. Our image generators take 2D axial-plane slices of the volumes as inputs. All volumes were resampled to a resolution of $1.8\times 1.8\times 1.8{\text{ mm}}^{3}∕voxel$, then cropped to a size of 128×128 pixels. As each resampled volume contains 94 to 102 slices, over 6000 pairs of IXI images were used in our experiments. As the generators in both CycleGAN and DiCyc are fully convolutional, the predictions are performed on uncropped images. All the images are bias field corrected and normalized with their mean and standard deviation.

${\mathrm{MA}}^{3}\mathrm{RS}$ **dataset:** We used a dataset containing 40 pairs of multi-modality abdominal T2*-weighted and CT images collected from 20 patients with abdominal aortic aneurysm. Example images are shown in Fig. 1 where domain-specific deformations can be observed. The data were collected as part of the $MA^{3}RS$ clinical trial^5^ [57]. All images were resampled to a resolution of $1.56\times 1.56\times 5{\text{ mm}}^{3}∕voxel$, and the axial-plane slices trimmed to 192×192 pixels. We used 30 volumes for training and 10 volumes for testing. Each resampled volume contains 24 to 40 slices, which gives over 1200 pairs of slices for our experiments.

### 4.2 Evaluation metrics

Ideally, alignment between data and the quality of the synthesized images can be evaluated by segmentation-based metrics, such as, Dice index. However, it is difficult to generate the segmentation masks on synthesized data, which can also introduce extra errors in the evaluation. Referring to previous image synthesis works discussed in previous sections, here we use three metrics to evaluate performance of image synthesis: mean squared error (MSE), peak signal-to-noise ratio (PSNR) and structural similarity index (SSIM) as typically used by other CycleGAN based methods. Given a volume $x^{A}$ and a target volume $x^{B}$, the MSE is computed as: $\frac{1}{N}\sum_{1}^{N}{\left(x^{B}-M^{A\to B}\left(x^{A}\right)\right)}^{2}$, where N is number of voxels in the volume. PSNR is calculated as: $10\log_{10}\frac{\max_{B}^{2}}{MSE}$, where $\max_{B}$ is the maximum voxel value of the image $x^{B}$. SSIM is computed as: $\frac{\left(2\mu_{A}\mu_{B}+c_{1}\right)\left(2\delta_{AB}+c2\right)}{\left(\mu_{A}^{2}+\mu_{B}^{2}+c_{1}\right)\left(\delta_{A}^{2}+\delta +B^{2}+c2\right)}$, where μ and $\delta^{2}$ are mean and variance of a volume, and $\delta_{AB}$ is the covariance between $x^{A}$ and $x^{B}$. $c_{1}$ and $c_{2}$ are two variables to stabilize the division with weak denominator [58]. Larger PSNR and SSIM, or smaller MSE, indicate a better performance of a synthesis algorithm. These metrics were used to identify the best performing CycleGAN-based method, which we will subsequently refer to as the baseline method. We then evaluated the performance of the proposed DiCyc method compared to this baseline method. A paired t-test was used to the difference in mean MSE, PSNR and SSIM values between DiCyc and selected baseline. For the ablation experiment, a paired t-test was performed on metrics arising from the DiCyc model and its CycleGAN-based counterpart. Differences in performance were considered to be statistically significant when the pvalue resulting from the t-test was less than 0.05.

### 4.3 Experimental setup

We present three experiments using the two datasets. In the first and second, performance of our DiCyc model was compared to the vanilla CycleGAN [28] and state-of-the-art CycleGAN models with image alignment losses [32], [48]. For all experiments, we applied random affine transformations, including translation, rotation, scaling, shearing and flipping, to the input data as augmentations in the training stage,^6^ and we manually set that each epoch contains 6000 iterations for better network convergence. After comparing performance of the proposed DiCyc with selected state-of-the-art methods, an ablation study was performed to reveal the influence of DiCyc architecture and learning procedure.

**Simulated IXI to identify influence of domain-specific deformation:** As the brain organs are mainly rigid structures and rarely suffer from non-linear deformations, ground truth obtained from the registered PD- and T2-weighted image pairs allows evident quantitative assessments. When trained on the registered data, all the methods obtained better performance than when they were trained on unaligned and unpaired data. This provided an upper limit of performance for all the tested methods. To assess the ability of the selected methods to deal with domain-specific deformations, we applied a simulated nonlinear transformation to each T2-weighted image. We performed synthesis experiments using the undeformed PD-weighted images and deformed T2-weighted images to generate undeformed T2-weighted data and deformed PD-weighted data. Minibatches of the input data were sampled from randomly selected patients and slices. When using deformed T2-weighted images to generate synthesized PD data, the ground truth was generated by applying the same nonlinear deformation to the source PD images. Similarly, the ground truth for the synthesized T2-weighted data were the original undeformed T2-weighted data provided in IXI. Values for the three evaluation metrics were computed between the synthesized images and the ground truths. We also qualitatively evaluate the synthesized images using error images as in prior works [26], [29].

${\mathrm{MA}}^{3}\mathrm{RS}$ **data:** After evaluated on simulated dataset with given ground truths, the methods are further evaluated using realistic data from our ${\mathrm{MA}}^{3}\mathrm{RS}$ dataset. Due to “domain-specific deformations”, the multi-modality images cannot be affinely registered. Specifically, the multiple organs in the pair of images can be hardly aligned at the same time. Furthermore, as non-rigid registration remains an ill-posed problem and lacks a gold standard, we did not non-rigidly register the images to generate ground truth for synthesis. However, several objects, such as aorta and spine, are relatively rigid compared to other surrounding soft tissues such as lower gastrointestinal tract organs. These objects can be separately registered with affine transformations. As a result, performance of synthesis should be assessed by alignment of multiple organs, as well as by quantitative analysis of image quality. In this work, for each volume in the $MA^{3}RS$ dataset, the anatomy of the aorta was manually segmented (as described in [59]). Multi-modality data acquired from the same patient were affinely registered so that the segmented aortas were well aligned. The manual registration and segmentation were performed by 4 clinical researchers. Signal of the synthesized images was evaluated within the segmentation of aorta using the three metrics described above. Image alignment between the source and synthesized data were visually assessed within both the aorta and spine regions. To sum up, a method with better performance should generate images show better alignment in both the aorta and spine region while achieving lower MSE, higher PSNR, and higher SSIM. In the training stage, the input minibatch was sampled from the same patient but randomly selected slices as described in [48]. The data is augmented with similar transformations that have been applied to the IXI dataset.

**Ablated models with different alignment losses:** The CycleGAN-based models do not handle the conflict between the additive image alignment losses and the discriminative GAN loss, thus cannot achieve good data alignment without sacrificing quality of the synthesized data. By contrast, the architecture and associated training algorithm of DiCyc handles the geometric deformation and contextual correspondence between the domains separately. This property plays a key role in generating synthesized data that are aligned with source data while maintaining a good performance of contextual synthesis. To prove this argument, it is necessary to analyze the different behaviors of an image alignment loss while being used in CycleGAN and DiCyc frameworks. Furthermore, current CycleGAN-based models use GCC and MIND, but we use a NMI-based alignment loss given in Eq. (19). To verify our proposed alignment loss, it is necessary to compare the performance GCC, MIND and NMI under the same architecture and training procedure.

With these motivations in mind, we performed an ablation experiment using the IXI dataset where different image alignment losses were integrated within both CycleGAN and DiCyc models. Specifically, we replaced NMI-based alignment loss used in the proposed model with the GCC- and MIND-based alignment loss to build a GCC-DiCyc and a MIND-DiCyc. Similarly, our NMI-based alignment loss was added to the CycleGAN loss to build a NMI-CycleGAN. Performance of DiCyc’s with different alignment losses were then compared to their CycleGAN-based counterparts. We performed a paired t-test on the evaluation metrics for each pair of CycleGAN and DiCyc models with the same alignment loss to evaluate any improvement in performance introduced by our new architecture. Any improvements introduced by the NMI-based alignment loss can be seen by comparing performance of the DiCyc models using different alignment losses. Evolution of the loss values and synthesis results were also visually assessed throughout the training process.

### 4.4 Implementation details

We used image generators with 6 Resnet blocks, and 70 × 70 PatchGAN [60] as discriminator networks. Based on the default setup of CycleGAN, we use the LSGAN loss to compute $L_{GAN}$. Experiments were implemented in PyTorch and paired t-tests were performed using Scipy library. All parameters of, or inherit from, vanilla CycleGAN are taken from the PyTorch implementation of the original paper.^7^ The first convolutional layer uses 7×7 kernels, all others use 3×3 kernels. The first convolution output 64 channels of feature maps, followed by layers with 128 and 256 channels. All the convolutions in the Resnet blocks have 256 channels.

For the DiCyc, we set $\lambda_{cyc}=\lambda_{dicyc}=10$ and $\lambda_{align}=0.9$. The models were trained with Adam optimizer [61] with a fixed learning rate of 0.0002 for the first 100 epochs, followed by 100 epochs with linearly decreasing learning rate. Here we apply a simple early stop strategy: in the first 100 epochs, when $L_{DiCyc}$ stops decreasing for 10 epochs, the training will move to the learning rate decaying stage; similarly, this tolerance is set to 20 epochs in the second 100 epochs. For the selected benchmark CycleGAN-based models, unless mentioned above, setup of hyper-parameters follows the original publications. Experiments were performed with nVidia Tesla K80 GPUs provided by the Amazon AWS EC2 cloud computing platform.

## 5 Results and discussion

This section presents the performance across all models assessed. For each experiment, we visualize the data from the source domain and the synthesized results. Quantitative results are shown in terms of MSE, PSNR and SSIM.

**Fig. 5:**
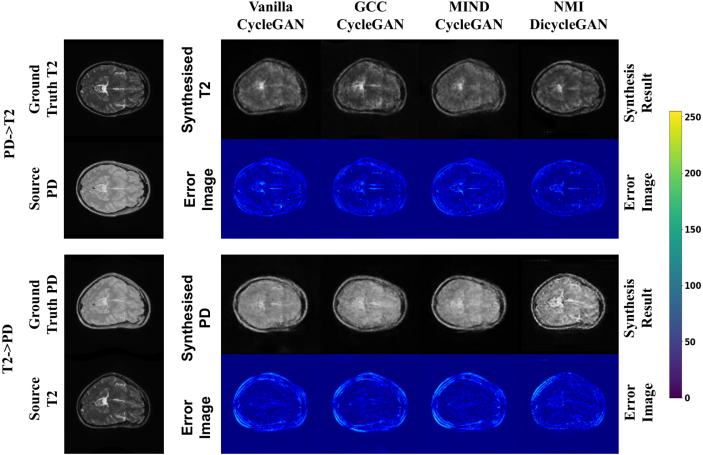
Examples of synthesis from the IXI dataset: an arbitrary deformation was applied to the T2 weighted images, and the ground truth of the synthesized proton density (PD) weighted image was generated by applying the same deformation.

### 5.1 DiCyc versus CycleGAN-based models on IXI

Fig. 5 shows an example of the synthesized images generated by the methods we tested, along with the error images calculated between the synthesized data and corresponding ground truth. For a fair visual comparison, here we present the results obtained by all the compared baselines with the same non-linear deformation. As the simulated “domain-specific deformation” were applied to the T2-weighted data, the synthesized PD-weighted data should display the same deformation aligned with the source data. Similarly, the synthesized T2-weighted data should be aligned with the source PD-weighted data without showing the simulated deformation. However, as shown in Fig. 5, the vanilla CycleGAN model reproduced the simulated deformation in the synthesized T2-weighted image and did not show the simulated deformation in the synthesized PD-weighted image. Although the GCC-CycleGAN and MIND-CycleGAN reduce the misalignment effect of the simulated deformation, the synthesized and source data are still not well aligned. Furthermore, the synthesis results generated by the three CycleGAN-based models are blurry and showed visible artifacts. In contrast, our DiCyc model gave the best alignment between the source and synthesized data and also lead to better image quality when assessed visually.

The quantitative evaluation of multi-sequence MR synthesis using the IXI dataset is shown in Table 1, where the best result for each metric is shown in bold and the optimum baseline method we chose for a paired t-test is highlighted by a gray background. Vanilla CycleGAN trained on paired and registered images (without simulated deformation) gave the best results with PSNR >24.3, SSIM >0.817 and MSE ≤0.036. This is considered as the upper bound of synthesis performance. Trained with unpaired data that have simulated deformations, the vanilla CycleGAN gave a lower-bound baseline of performance. With additive image alignment losses, GCC-CycleGAN and MIND-CycleGAN methods lead to improvements in terms of PSNR. However, because these two models are still affected by the simulated domain-specific deformation, their performance was still comparable to vanilla CycleGAN.

In contrast, the proposed DiCyc model led to at least 18% increase in MSE, and 8% and 12% performance gain in terms of PSNR and SSIM on IXI data. The results were statistically significant based on the paired t-tests (p-value <0.05).

**Table 1:**
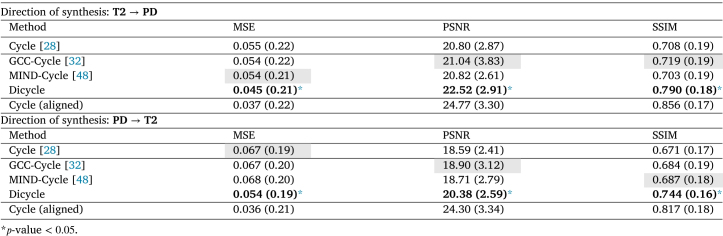
Synthesis results of IXI dataset using deformed T2 images given by value of each metric. Standard deviations are shown within parentheses.

### 5.2 DiCyc versus CycleGAN-based models on ${\mathrm{MA}}^{3}\mathrm{RS}$

Table 2 shows the quantitative assessments of the four models based on the same metrics used for the IXI data. The vanilla CycleGAN had slightly better performance compared to the GCC- and MIND-CycleGAN models. The only exception is that MNID-CycleGAN model obtained higher PSNR in the “T2*→CT” synthesis. Our DiCyc model outperformed the other three methods according to all the metrics. Note that in the “CT→T2*” synthesis, DiCyc lead to a 20% performance gain in terms of MSE, and achieved 22.8% higher SSIM. Differences between performance achieved by the DiCyc model and the best baseline methods were statistically significant.

The quantitative results shown in Table 2 can be affected by both the qualities of the synthesized images and the alignment between the source and synthesized data. As discussed above, some objects in the images can be affinely registered independently, for example, the anatomy of aorta and spine. However, these two objects cannot be affinely aligned at the same time as a result of domain-specific deformations. This leads to lower PSNR and SSIM, and higher MSE value within the segmented region of aorta.

**Fig. 6:**
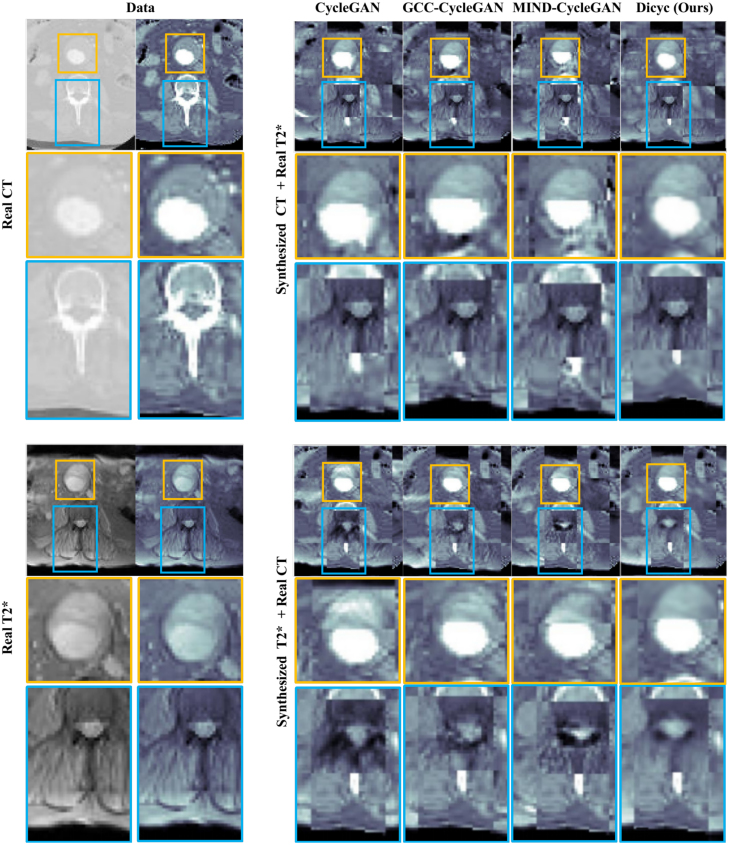
Visualization results on MA3RS data: the source and the associated synthesized images are displayed using a chessboard visualization. The regions of aorta and spine are highlighted by yellow and blue boxes. CycleGAN-based methods tend to reproduce the domain-specific deformation or suffer from significant artifacts. (For interpretation of the references to color in this figure legend, the reader is referred to the web version of this article.)

 For better assessing the effects of the domain-specific deformation, the synthesis results of the compared baselines and our TPS-based DiCyc model are displayed in Fig. 6 using a checkerboard visualization. As shown in Fig. 6, when the region of aorta is affinely aligned, the CycleGAN-based methods either achieved worse alignment in the spine area, for example, the synthesized CT produced by CycleGAN and GCC-CycleGAN, and the synthesized T2* weighted image given by GCC-CycleGAN; or they generated significant artifacts, for example, in the aorta area of synthesized CT output by CycleGAN and MIND-CycleGAN. Our DiCyc model is the only model that produces synthesized images where both the aorta and spine are simultaneously aligned. Although the synthesized T2* weighted images looks slightly blurred, our DiCyc model generated less artifacts.

**Table 2:**
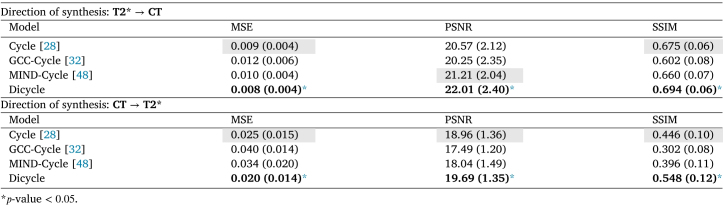
Multi-modality synthesis results using private dataset given by value of each metric. Standard deviations are shown within parentheses.

**Fig. 7:**
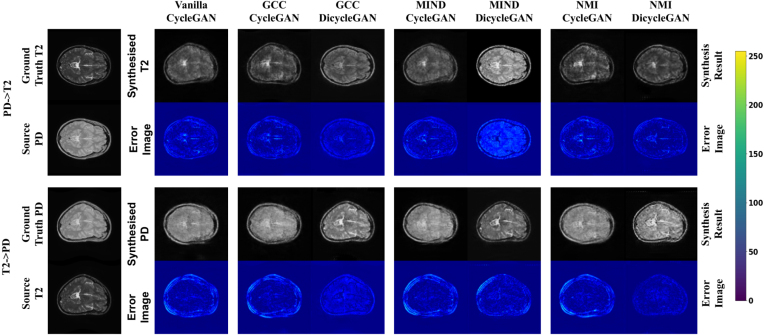
Visualization of results of ablated models with different image alignment losses. The results were obtained from the IXI dataset with the same simulated deformation applied to the PD-weighted MRI data. The difference image for each method is shown under the synthesis result it generated.

**Fig. 8:**
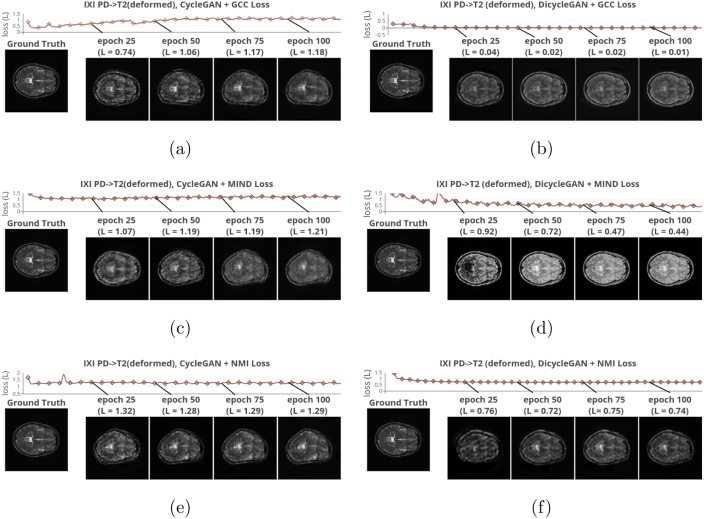
Evolution of synthesized data during the training process. 8a to 8f successively display the loss curves of GCC-CycleGAN [32], GCC-DiCyc, MIND-CycleGAN [48], MIND-DiCyc, NMI-CycleGAN and NMI-DiCyc (proposed). synthesized T2 weighted data obtained at the 25th, 50th, 75th, 100th epochs are shown above the curves, in comparison of the ground truth shown at the bottom right. (Best viewed in color).

**Fig. 9:**
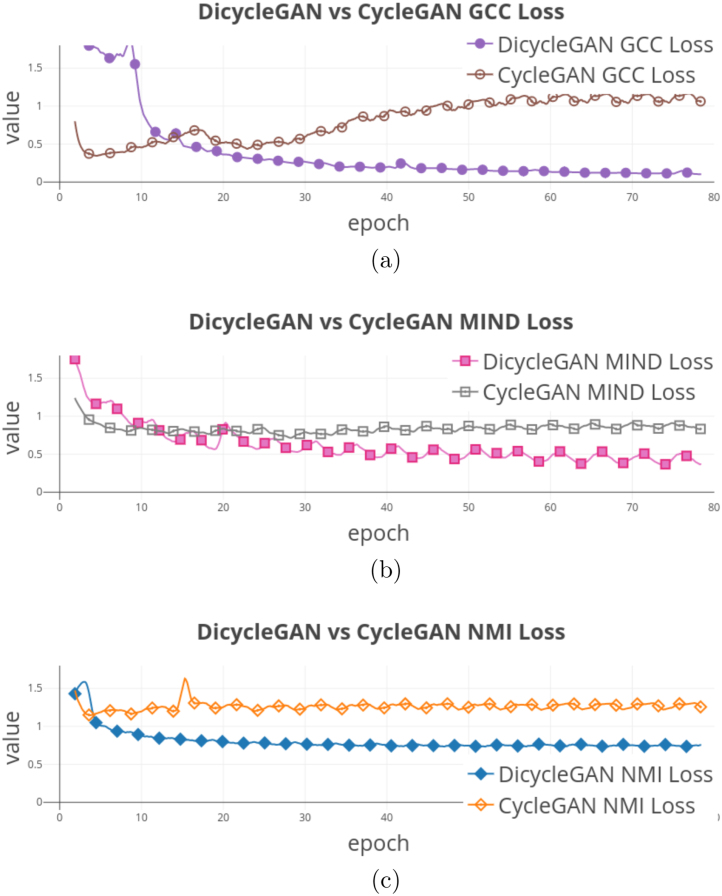
Evolution processes of each alignment loss in the training process while used in the CycleGAN and DiCyc framework: 9a GCC Loss, 9b MIND Loss, and 9c NMI Loss. (best viewed in color).

### 5.3 Ablation study

Fig. 7 presents the synthesized images produced by the ablated models using different alignment losses, and the quantitative evaluation results are shown in Table 3. As shown in Fig. 7, all the DiCyc-based models achieved better alignment between the source and synthesized data. This is consistent with the quantitative results shown in Table 3 where in most cases ablated DiCyc models achieved lower MSE and higher PSNR and SSIM models. However, using GCC- and MIND-based alignment losses within the DiCyc framework caused a shift of intensities in the synthesized data. The most obvious example is the synthesized T2-weighted image produced by MIND-DiCyc which looks more like the source PD-weighted data rather than the target T2-weighted data. As a result, the MIND-DiCyc model gave higher MSE and lower PSNR values in the “PD→T2” synthesis. By contrast, this intensity shift was not observed in the synthesized data generated by our proposed NMI-based DiCyc model. The proposed NMI-DiCyc model outperformed the ablated GCC-DiCyc and MIND-DiCyc models, as well as the state-of-the-art CycleGAN-based methods.

Fig. 8, Fig. 9 demonstrate the evolution of the compared image alignment losses and the synthesis results in the CycleGAN and DiCyc frameworks during the training process. Comparing the synthesis results produced by CycleGAN-based methods (Figs. 8a, 8c and 8e) with those generated by DiCyc models (Figs. 8b, 8d and 8f), we can see that the CycleGAN methods can achieve a good data alignment within the first 20 epochs of training. However as the training algorithm continues to minimize the CycleGAN losses, the domain-specific deformation is gradually reproduced. As the DiCyc framework separately trains the image alignment loss and the CycleGAN loss in two forward passes, the relative deformation between the source and target domain is removed. As shown in Figs. 9a, 9b and 9c, in the CycleGAN framework, each alignment loss was minimized at a certain point of the training process, but then kept increasing as it started to conflict with the GAN discriminative losses. In our DiCyc framework, the alignment losses kept decreasing throughout the whole training process.

**Table 3:** Multi-modality synthesis results using private dataset given by value of each metric. Standard deviations are shown within parentheses.

Direction of synthesis: **PD**→**T2**			
Model	MSE	PSNR	SSIM
GCC-Cycle [32]	0.054 (0.021)	21.04 (3.83)	0.719 (0.19)
GCC-Dicycle	**0.047 (0.006)**⁎	**22.17 (3.05)**⁎	**0.840 (0.18)**⁎

MIND-Cycle [48]	**0.054 (0.21)**	**20.82 (2.61)**	0.703 (0.19)
MIND-Dicycle	0.090 (0.22)⁎	18.59 (2.04)⁎	**0.714 (0.20)**⁎

NMI-Cycle	0.055 (0.22)	21.03 (3.06)	0.712 (0.20)
Dicycle (NMI)	**0.045 (0.21)**⁎	**22.52 (2.91)**⁎	**0.790 (0.18)**⁎

Direction of synthesis: **T2**→**PD**			

Model	MSE	PSNR	SSIM

GCC-Cycle [32]	0.067 (0.20)	18.90 (3.12)	0.684 (0.19)
GCC-Dicycle	**0.054 (0.20)**⁎	**20.42 (3.18)**⁎	**0.740 (0.20)**⁎

MIND-Cycle [48]	0.068 (0.20)	18.71 (2.79)	0.687 (0.18)
MIND-Dicycle	**0.054 (0.19)**⁎	**20.33 (2.97)**⁎	**0.740 (0.19)**⁎

NMI-Cycle	0.067 (0.21)	18.76 (3.09)	0.684 (0.19)
Dicycle (NMI)	**0.054 (0.19)**⁎	**20.38 (2.59)**⁎	**0.744 (0.19)**⁎

* p-value <0.05.

Comparing the results shown in Figs. 8b, 8d and 8f, we can see that the ablated GCC- and MIND-DiCyc models reproduced the appearance of the PD-weighted data in the synthesized T2-weighted data. This means GCC and MIND are still more domain-dependent measures compared to NMI although they have been widely used in multi-modality registration methods. However, computationally GCC and MIND are easily vectorized and the associated backward pass are easier to implement with lesser computational complexities.

### 5.4 Model complexity

For the CycleGAN-based baselines compared above, each generator network, M, has 34.52M trainable parameters, and each descriminator network, D, has 2.76M. As a result, in the training stage, a CycleGAN-based model has 74.56M trainable parameters and each forward pass consists of 37.98G multiply-add operations (MACs)^8^ processing 128 × 128 image data. For our DiCyc model, the local and the global transformation modules introduce 8.15M and 4.31M trainable parameters. Each forward pass consists of 66.36G MACs. As a result, it takes 75% more time and 33% extra memory to train a DiCyc model. However, once trained, prediction of the synthesized images is performed only by the image generator without global and local deformation modules. In other word, in the testing stage, the proposed DiCyc model has the same temporal and spacial complexity with the CycleGAN-based methods (34.52M trained parameters, 18.24G MACs per forward pass).

## 6 Conclusion

We introduced the DiCyc cross-domain medical image synthesis model which addresses the issue of and is resilient to domain-specific deformations. We integrated a modified deformable convolutional layer into the network architecture, and proposed the associateddeformation-invariant cycle consistency loss and NMI-based alignment loss function. Experiments were performed for synthesis of multi-sequence MRI data with simulated deformations and of multi-modality CT and MRI data suffering from actual domain-specific deformations. We compared our method to the vanilla CycleGAN method and two state-of-the-art methods with additional alignment losses. Our DiCyc method achieved better alignment between the source and synthesized data while maintaining signal qualities of the synthesized data. It outperformed state-of-the-art methods. In order to reveal the mechanism of DiCyc that is separately encoding the information about spatial deformation in the synthesis process, we also performed an ablation study by integrating popular image similarity metrics into DiCyc and comparing their CycleGAN-based counterparts. It shows that the DiCyc model avoids the conflict between the CycleGAN loss and the image alignment losses. Our NMI-based image alignment loss also demonstrated better robustness for synthesis of images from different domains.

## CRediT authorship contribution statement

**Chengjia Wang:** Conceptualization, Methodology, Software, Data curation, Writing - original draft, Visualization, Investigation, Writing - review & editing. **Guang Yang:** Conceptualization, Investigation, Visualization, Investigation, Writing - review & editing, Validation. **Giorgos Papanastasiou:** Methodology, Data curation, Writing - review & editing, Software, Investigation, Validation. **Sotirios A. Tsaftaris:** Methodology, Validation, Writing - review & editing. **David E. Newby:** Supervision, Validation. **Calum Gray:** Software, Data curation, Visualization, Methodology. **Gillian Macnaught:** Conceptualization, Data curation, Validation, Software, Writing - review & editing, Supervision. **Tom J. MacGillivray:** Conceptualization, Investigation, Validation, Software, Writing - review & editing, Supervision.

## Declaration of Competing Interest

The authors declare that they have no known competing financial interests or personal relationships that could have appeared to influence the work reported in this paper.

## Footnotes

2 This is also addressed as “image translation” in computer vision.

3 Here we set $\lambda_{cyc}=\lambda_{dicyc}=10$ and $\lambda_{align}=0.9$.

4 http://brain-development.org/ixi-dataset/.

5 http://www.isrctn.com/ISRCTN76413758.

6 The affine transformation are randomly generated within $\left[-15,15\right]$ translation, $\left[-15{}^{\circ},15{}^{\circ}\right]$ rotation, $\left[0.9,1.1\right]$ scaling, $\left[0.9,1.1\right]$ shearing, and random flip with a probability of 0.2. This setup makes sure the affine transformation does not move significant about of the imaged object out of the field of view so that quantitatively assessible results can be obtained..

7 https://github.com/junyanz/pytorch-CycleGAN-and-pix2pix.

8 1G multiply-add operation (MACs) is roughly 2G floating points operations (FLOPs). Results are obtained using the ptflops package at https://github.com/sovrasov/flops-counter.pytorch and the torchsummary package at https://github.com/sksq96/pytorch-summary.

## References

[1] van Tulder G., de Bruijne M. (2015). Why does synthesized data improve multi-sequence classification?. International Conference on Medical Image Computing and Computer-Assisted Intervention.

[2] Dalca A.V., Bouman K.L., Freeman W.T., Rost N.S., Sabuncu M.R., Golland P. (2018). Medical image imputation from image collections. IEEE Trans. Med. Imaging.

[3] Eilertsen K., Nilsen Tor Arne Vestad L., Geier O., Skretting A. (2008). A simulation of MRI based dose calculations on the basis of radiotherapy planning CT images. Acta Oncol..

[4] Iglesias J.E., Konukoglu E., Zikic D., Glocker B., Van Leemput K., Fischl B. (2013). Is synthesizing MRI contrast useful for inter-modality analysis?. International Conference on Medical Image Computing and Computer-Assisted Intervention.

[5] Du J., Li W., Lu K., Xiao B. (2016). An overview of multi-modal medical image fusion. Neurocomputing.

[6] He Q., Li X., Kim D.N., Jia X., Gu X., Zhen X., Zhou L. (2020). Feasibility study of a multi-criteria decision-making based hierarchical model for multi-modality feature and multi-classifier fusion: Applications in medical prognosis prediction. Inf. Fusion.

[7] Wang K., Zheng M., Wei H., Qi G., Li Y. (2020). Multi-modality medical image fusion using convolutional neural network and contrast pyramid. Sensors.

[8] Roy S., Carass A., Prince J. (2011). A compressed sensing approach for MR tissue contrast synthesis. Biennial International Conference on Information Processing in Medical Imaging.

[9] Chartsias A., Joyce T., Papanastasiou G., Semple S., Williams M., Newby D., Dharmakumar R., Tsaftaris S.A. (2018). Factorised spatial representation learning: application in semi-supervised myocardial segmentation. International Conference on Medical Image Computing and Computer-Assisted Intervention.

[10] Li L., Zhao X., Lu W., Tan S. (2020). Deep learning for variational multimodality umor segmentation in pet/ct. Neurocomputing.

[11] Cordier N., Delingette H., Lê M., Ayache N. (2016). Extended modality propagation: image synthesis of pathological cases. IEEE Trans. Med. Imaging.

[12] Commowick O., Warfield S.K., Malandain G. (2009). Using frankensteins creature paradigm to build a patient specific atlas. International Conference on Medical Image Computing and Computer-Assisted Intervention.

[13] Li R., Zhang W., Suk H.-I., Wang L., Li J., Shen D., Ji S. (2014). Deep learning based imaging data completion for improved brain disease diagnosis. International Conference on Medical Image Computing and Computer-Assisted Intervention.

[14] Zhou T., Thung K.-H., Liu M., Shi F., Zhang C., Shen D. (2020). Multi-modal latent space inducing ensemble svm classifier for early dementia diagnosis with neuroimaging data. Med. Image Anal..

[15] Wagenknecht G., Kaiser H.-J., Mottaghy F.M., Herzog H. (2013). MRI for attenuation correction in pet: methods and challenges. Magn. Reson. Mater. Phys. Biol. Med..

[16] Torrado-Carvajal A., Herraiz J.L., Alcain E., Montemayor A.S., Garcia-Cañamaque L., Hernandez-Tamames J.A., Rozenholc Y., Malpica N. (2016). Fast patch-based pseudo-CT synthesis from T1-weighted MR images for PET/MR attenuation correction in brain studies. J. Nucl. Med..

[17] Burgos N., Cardoso M.J., Thielemans K., Modat M., Pedemonte S., Dickson J., Barnes A., Ahmed R., Mahoney C.J., Schott J.M. (2014). Attenuation correction synthesis for hybrid PET-MR scanners: application to brain studies. IEEE Trans. Med. Imaging.

[18] Gong K., Yang J., Kim K., El Fakhri G., Seo Y., Li Q. (2018). Attenuation correction for brain PET imaging using deep neural network based on Dixon and ZTE MR images. Phys. Med. Biol..

[19] Roy S., Wang W.-T., Carass A., Prince J.L., Butman J.A., Pham D.L. (2014). PET attenuation correction using synthetic CT from ultrashort echo-time MR imaging. J. Nucl. Med..

[20] Leynes A.P., Yang J., Wiesinger F., Kaushik S.S., Shanbhag D.D., Seo Y., Hope T.A., Larson P.E. (2018). Zero-echo-time and dixon deep pseudo-ct (zedd ct): direct generation of pseudo-ct images for pelvic pet/mri attenuation correction using deep convolutional neural networks with multiparametric mri. J. Nucl. Med..

[21] Bowles C., Qin C., Ledig C., Guerrero R., Gunn R., Hammers A., Sakka E., Dickie D.A., Hernández M.V., Royle N. (2016). Pseudo-healthy image synthesis for white matter lesion segmentation. International Workshop on Simulation and Synthesis in Medical Imaging.

[22] Chartsias A., Joyce T., Giuffrida M.V., Tsaftaris S.A. (2017). Multimodal MR synthesis via modality-invariant latent representation. IEEE Trans. Med. Imaging.

[23] Cohen J.P., Luck M., Honari S. (2018). Distribution matching losses can hallucinate features in medical image translation. International Conference on Medical Image Computing and Computer-Assisted Intervention.

[24] Johansson A., Garpebring A., Asklund T., Nyholm T. (2014). CT substitutes derived from MR images reconstructed with parallel imaging. Med. Phys..

[25] Joyce T., Chartsias A., Tsaftaris S.A. (2017). Robust multi-modal mr image synthesis. International Conference on Medical Image Computing and Computer-Assisted Intervention.

[26] Nie D., Trullo R., Lian J., Petitjean C., Ruan S., Wang Q., Shen D. (2017). Medical image synthesis with context-aware generative adversarial networks. International Conference on Medical Image Computing and Computer-Assisted Intervention.

[27] Van Nguyen H., Zhou K., Vemulapalli R. (2015). Cross-domain synthesis of medical images using efficient location-sensitive deep network. International Conference on Medical Image Computing and Computer-Assisted Intervention.

[28] Wolterink J.M., Dinkla A.M., Savenije M.H., Seevinck P.R., van den Berg C.A., Išgum I. (2017). Deep MR to CT synthesis using unpaired data. International Workshop on Simulation and Synthesis in Medical Imaging.

[29] Nie D., Trullo R., Lian J., Wang L., Petitjean C., Ruan S., Wang Q., Shen D. (2018). Medical image synthesis with deep convolutional adversarial networks. IEEE Trans. Biomed. Eng..

[30] Huo Y., Xu Z., Bao S., Assad A., Abramson R.G., Landman B.A. (2018). Adversarial synthesis learning enables segmentation without target modality ground truth. Biomedical Imaging (ISBI 2018), 2018 IEEE 15th International Symposium on.

[31] Chartsias A., Joyce T., Dharmakumar R., Tsaftaris S.A. (2017). Adversarial image synthesis for unpaired multi-modal cardiac data. International Workshop on Simulation and Synthesis in Medical Imaging.

[32] Hiasa Y., Otake Y., Takao M., Matsuoka T., Takashima K., Carass A., Prince J.L., Sugano N., Sato Y. (2018). Cross-modality image synthesis from unpaired data using cyclegan. International Workshop on Simulation and Synthesis in Medical Imaging.

[33] Costa P., Galdran A., Meyer M.I., Niemeijer M., Abràmoff M., Mendonça A.M., Campilho A. (2017). End-to-end adversarial retinal image synthesis. IEEE Trans. Med. Imaging.

[34] R. Vemulapalli, H. Van Nguyen, S. Kevin Zhou, Unsupervised cross-modal synthesis of subject-specific scans, in: Proceedings of the IEEE International Conference on Computer Vision, 2015, pp. 630–638.

[35] Goodfellow I., Pouget-Abadie J., Mirza M., Xu B., Warde-Farley D., Ozair S., Courville A., Bengio Y. (2014). Generative adversarial nets. Advances in Neural Information Processing Systems.

[36] Lan L., You L., Zhang Z., Fan Z., Zhao W., Zeng N., Chen Y., Zhou X. (2020). Generative adversarial networks and its applications in biomedical informatics. Front. Public Health.

[37] J.-Y. Zhu, T. Park, P. Isola, A.A. Efros, Unpaired image-to-image translation using cycle-consistent adversarial networks, in: Proceedings of the IEEE International Conference on Computer Vision, 2017, pp. 2223–2232.

[38] Iglesias J.E., Modat M., Peter L., Stevens A., Annunziata R., Vercauteren T., Lein E., Fischl B., Ourselin S., Initiative A.D.N. (2018). Joint registration and synthesis using a probabilistic model for alignment of MRI and histological sections. Med. Image Anal..

[39] Jog A., Carass A., Roy S., Pham D.L., Prince J.L. (2017). Random forest regression for magnetic resonance image synthesis. Med. Image Anal..

[40] Martinez-Murcia F.J., Górriz J.M., Ramírez J., Illán I.A., Segovia F., Castillo-Barnes D., Salas-Gonzalez D. (2017). Functional brain imaging synthesis based on image decomposition and kernel modeling: Application to neurodegenerative diseases. Front. Neuroinform..

[41] Ye D.H., Zikic D., Glocker B., Criminisi A., Konukoglu E. (2013). Modality propagation: coherent synthesis of subject-specific scans with data-driven regularization. International Conference on Medical Image Computing and Computer-Assisted Intervention.

[42] Jog A., Roy S., Carass A., Prince J.L. (2013). Magnetic resonance image synthesis through patch regression. Biomedical Imaging (ISBI), 2013 IEEE 10th International Symposium on.

[43] Jog A., Carass A., Roy S., Pham D.L., Prince J.L. (2015). MR image synthesis by contrast learning on neighborhood ensembles. Med. Image Anal..

[44] N. Bayramoglu, M. Kaakinen, L. Eklund, J. Heikkila, Towards virtual h&e staining of hyperspectral lung histology images using conditional generative adversarial networks, in: Proceedings of the IEEE International Conference on Computer Vision Workshops, 2017, pp. 64–71.

[45] Huang Y., Shao L., Frangi A.F. (2018). Cross-modality image synthesis via weakly coupled and geometry co-regularized joint dictionary learning. IEEE Trans. Med. Imaging.

[46] Zhu Y., Tang Y., Tang Y., Elton D.C., Lee S., Pickhardt P.J., Summers R.M. (2020). Cross-domain medical image translation by shared latent Gaussian mixture model. arxiv:2007.07230.

[47] Liu M.-Y., Breuel T., Kautz J., Guyon I., Luxburg U.V., Bengio S., Wallach H., Fergus R., Vishwanathan S., Garnett R. (2019). Unsupervised image-to-image translation networks. Advances in Neural Information Processing Systems 30.

[48] Yang H., Sun J., Carass A., Zhao C., Lee J., Xu Z., Prince J. (2018). Unpaired brain MR-to-CT synthesis using a structure-constrained cyclegan. Deep Learning in Medical Image Analysis and Multimodal Learning for Clinical Decision Support.

[49] Heinrich M.P., Jenkinson M., Bhushan M., Matin T., Gleeson F.V., Brady M., Schnabel J.A. (2012). MIND: Modality independent neighbourhood descriptor for multi-modal deformable registration. Med. Image Anal..

[50] Wang C., Papanastasiou G., Tsaftaris S., Yang G., Gray C., Newby D., Macnaught G., MacGillivray T. (2019). Tpsdicyc: Improved deformation invariant cross-domain medical image synthesis. International Workshop on Machine Learning for Medical Image Reconstruction.

[51] Qin C., Shi B., Liao R., Mansi T., Rueckert D., Kamen A. (2019). Unsupervised deformable registration for multi-modal images via disentangled representations. International Conference on Information Processing in Medical Imaging.

[52] Chartsias A., Joyce T., Papanastasiou G., Semple S., Williams M., Newby D.E., Dharmakumar R., Tsaftaris S.A. (2019). Disentangled representation learning in cardiac image analysis. Med. Image Anal..

[53] Kent J., Mardia K. (1994). The link between kriging and thin-plate splines.

[54] Dai J., Qi H., Xiong Y., Li Y., Zhang G., Hu H., Wei Y. (2017). Deformable convolutional networks. arxiv:abs/1703.06211.

[55] Chen X., Duan Y., Houthooft R., Schulman J., Sutskever I., Abbeel P. (2016). Infogan: Interpretable representation learning by information maximizing generative adversarial nets. Advances in Neural Information Processing Systems.

[56] Vinh N.X., Epps J., Bailey J. (2010). Information theoretic measures for clusterings comparison: Variants, properties, normalization and correction for chance. J. Mach. Learn. Res..

[57] Newby D., Forsythe R., McBride O., Robson J., Vesey A., Chalmers R., Burns P., Garden O.J., Semple S. (2017). Aortic wall inflammation predicts abdominal aortic aneurysm expansion, rupture, and need for surgical repair. Circulation.

[58] Hore A., Ziou D. (2010). Image quality metrics: PSNR vs. SSIM. Pattern Recognition (ICPR), 2010 20th International Conference on.

[59] Papanastasiou G., González-Castro V., Gray C., Forsythe R., Sourgia-Koutraki Y., Mitchard N., Newby D.E., Semple S. (2017). Multidimensional assessments of abdominal aortic aneurysms by magnetic resonance against ultrasound diameter measurements. Annual Conference on Medical Image Understanding and Analysis.

[60] P. Isola, J.-Y. Zhu, T. Zhou, A.A. Efros, Image-to-image translation with conditional adversarial networks, in: Proceedings of the IEEE Conference on Computer Vision and Pattern Recognition, 2017, pp. 1125–1134.

[61] Kingma D.P., Ba J. (2015). Adam: A method for stochastic optimization. International Conference on Learning Representations (ICLR).

